# A Comprehensive Evaluation of Tomato Fruit Quality and Identification of Volatile Compounds

**DOI:** 10.3390/plants12162947

**Published:** 2023-08-15

**Authors:** Jing Zhang, Sitian Liu, Xiumei Zhu, Youlin Chang, Cheng Wang, Ning Ma, Junwen Wang, Xiaodan Zhang, Jian Lyu, Jianming Xie

**Affiliations:** 1 College of Horticulture, Gansu Agricultural University, Anning District, Yingmeng Village, Lanzhou 730070, China; zj@gsau.edu.cn (J.Z.); liust0119@163.com (S.L.); changyoulin98@163.com (Y.C.); GSAUPHD0810@outlook.com (C.W.); MaNing96@139.com (N.M.); wangjw0314@163.com (J.W.); zhangxiaod@gsau.edu.cn (X.Z.); lvjian@gsau.edu.cn (J.L.); 2 Gansu Inspection and Testing Center for Agricultural Product Quality and Safety, Lanzhou 730000, China; beckyxiumei@163.com

**Keywords:** tomato, flavor, quality evaluation, volatile compounds

## Abstract

Tomatoes (*Lycopersicon esculentum*) are the most valuable vegetable crop in the world. This study identified the morphological characteristics, vitamin content, etc., from 15 tomato varieties in total, that included five each from the three experimental types, during the commercial ripening period. The results showed that the hardness with peel and the moisture content of tasty tomatoes were 157.81% and 54.50%, and 3.16% and 1.90% lower than those of regular tomatoes and cherry tomatoes, respectively, while the soluble solids were 60.25% and 20.79% higher than those of the latter two types. In addition, the contents of vitamin C, lycopene, fructose, glucose, and total organic acids of tasty tomatoes were higher than those of regular tomatoes and cherry tomatoes. A total of 110 volatile compounds were detected in the 15 tomato varieties. The average volatile compound content of tasty tomatoes was 57.94% higher than that of regular tomatoes and 15.24% higher than that of cherry tomatoes. Twenty of the 34 characteristic tomato aroma components were identified in tasty tomatoes, with fruity and green being the main odor types. Ten characteristic aroma components in regular tomatoes were similar to those of tasty tomatoes; ten types of cherry tomatoes had floral and woody aromas as the main odor types. The flavor sensory score was significantly positively correlated with the content of soluble solids, fructose, glucose, citric acid, fumaric acid, and β-ionone (*p* < 0.01), and significantly negatively correlated with water content and firmness without peel. Regular, tasty, and cherry tomatoes were separated using principal component analysis, and the quality of tasty tomatoes was found to be better than cherry tomatoes, followed by regular tomatoes. These results provide valuable information for a comprehensive evaluation of fruit quality among tomato varieties to develop consumer guidelines.

## 1. Introduction

Tomato (*Lycopersicon esculentum*) belongs to the Solanaceae family. It is native to South America and is one of the most widely consumed fruit crops in the world [1,2]. It can be eaten fresh and cooked, is rich in a variety of nutrients, vitamins, and mineral elements, and this motivates its frequent use as a model species to study nutritional metabolism [3,4,5]. The global tomato planting area was 5.05 million hm^2^ in 2020, and the output reached 187 million tons, with the total output value exceeding 60 billion dollars. Currently, China is the world’s largest tomato-producing and -consuming country (United Nations Food and Agriculture Organization statistics, 2020).

The qualities of tomatoes include their appearance, flavor, nutrition, processing and storage characteristics, among others [6,7]. In recent years, cultivation of tomato varieties has been focused on enhanced firmness to ensure long-distance storage and transportation. However, tomatoes generally have low requirements for firmness to achieve a good taste [8]. Vitamin C has a strong antioxidant effect and is an important nutrient that determines tomato quality. The external environment (such as light, water, and fertilizer conditions) plays an important role in regulating vitamin C synthesis [9,10]. Flavor is the main factor affecting consumer preference. This largely depends on the content of soluble solids, sugar, and organic acids. Soluble solids is the general term for soluble compounds in tomato fruit that directly affect the taste [11,12]. The sweet taste of tomato is mainly derived from fructose and glucose, while the sour taste is mainly attributed to citric and malic acids [13]. Therefore, the sugar and acid contents are often used as important indicators for evaluating tomato flavor. However, under the same cultivation conditions, genetic factors mainly control the formation of flavor substances.

Aroma is composed of a variety of volatile compounds and is an important factor that determines tomato fruit flavor [14]. Volatile compounds have the characteristics of low melting point, high vapor pressure, small molecular weight, and low content. The combination of these molecules with sugars and acids enhances tomato flavor, determines the uniqueness of tomato flavor [15], affects consumers’ sensory choice and acceptance of tomato products [16], and even plays a decisive role in consumer preference and purchase desire to choose tomatoes [17,18]. Research on plant volatile compounds has gradually increased with the development of identification technology such as gas chromatography–mass spectrometry (GC-MS). At present, over 400 volatile compounds have been identified in tomato fruit [19]. However, most current research focuses on a single variety, with few studies on different types of tomato.

The market demand for high-quality agricultural products has diversified with increased consumption levels; tomatoes with different quality characteristics are, thus, being cultivated. In particular, cherry tomato fruit differs in fruit shape and color and contains rich mineral elements, vitamin C, and a variety of nutrients that are typically present in a higher content than in regular tomatoes (except water content) [20]. In addition, unlike regular tomatoes, a type of large fruit tomato with a thin skin, soft meat, high soluble solids content, and good flavor is called the tasty tomato. However, there is a lack of detailed quality information on the tasty tomato, and a comparative analysis of the fruit quality among regular, cherry, and tasty tomatoes has not been reported. Therefore, as the planting area of tomatoes expands year by year and the variety of types continues to increase, establishing quantifiable evaluation indicators for the agronomic traits and quality of the tasty tomato, and constructing a comprehensive evaluation system for different types of tomatoes has become an urgent problem to be solved. This study compared the differences in fruit shape and color, and the contents of soluble solids, sugars, organic acids, vitamin C, volatile compounds, and fruit quality of the regular tomato, tasty tomato, and cherry tomato. Furthermore, the quality characteristics of different types of tomato and the correlation between volatile compounds and sensory evaluation were studied. The results are expected to provide a theoretical basis for quality tomato breeding and lead to further improvements in tomato quality.

## 2. Materials and Methods

### 2.1. Plant Material

**Figure 1:**
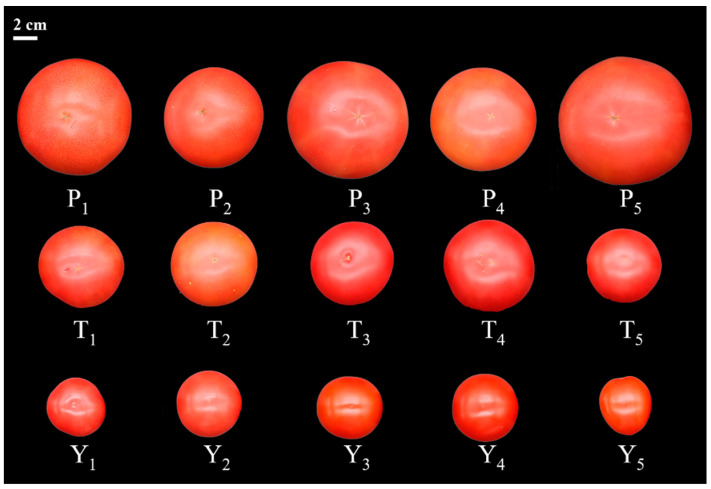
Tomato fruit varieties used in this study: P_1_, Huachen 70; P_2_, Yinfeng 79; P_3_, Puluowangsi; P_4_, Hunshuo 188; P_5_, Dongfeng 199; T_1_, Zhengfan P190; T_2_, Zhengfan P210; T_3_, Youyou 2045; T_4_, Gaotang 100; T_5_, Yuanwei No.1; Y_1_, Pink Baby; Y_2_, Bay Butter; Y_3_, Red Bean; Y_4_, Hongmei 1850; Y_5_, Juan-zhulian.

The 15 tomato varieties, including 5 regular tomatoes (P_1_, Huachen 70; P_2_, Yinfeng 79; P_3_, Puluowangsi; P_4_, Hunshuo 188; P_5_, Dongfeng 199), 5 tasty tomatoes (T_1_, Zhengfan P190; T_2_, Zhengfan P210; T_3_, Youyou 2045; T_4_, Gaotang 100; T_5_, Yuanwei No. 1), and 5 cherry tomatoes (Y_1_, Pink Baby; Y_2_, Bay Butter; Y_3_, Red Bean; Y_4_, Hongmei 1850; Y_5_, Juan-zhulian) were grown at the solar greenhouse of Gobi Agricultural Industrial Park, Suzhou District, Jiuquan City, China (Figure 1). All tomato materials were locally grown on a large scale, are popular among consumers, and were planted in July 2021 in a north–south orientation, with a row spacing of 0.8–1 m. Fertilization management and pest control were carried out according to the standard practices of the industrial park. A total of 300 fruits, at the commercial maturity stage, were picked during the harvest season (from the 12 to the 30 of January in 2022) based on uniformity, external color, and size for each variety. The fruits were immediately sent to the laboratory following the harvest. The peduncle was removed, the tomato samples were washed (first with running water, and then with distilled water), and residual moisture was evaporated at room temperature.

### 2.2. Measurement of Fruit Physical Parameters

Nine tomato fruits of each variety were randomly selected to determine the fruit firmness, shape index, and moisture content of the tomato variety.

The longitudinal and transverse diameters of the fruit were measured with a vernier caliper, and the vertical direction was re-measured. The fruit shape index was calculated from the ratio of the longitudinal and transverse diameters of the fruit [21].

The fruit firmness of each tomato was measured at two points of the equatorial region using the GY-4-J fruit pressure tester (Zhejiang Topuyunnong Technology Co., Ltd., Zhejiang, China). The probe penetrated the sample with a uniform force to a depth of 10 mm and provided a pressure measurement expressed in kg/cm. The measurement was conducted before peeling and after removing 1 mm of peel with a paring knife.

An analytical balance was used to weigh the fresh weight of a quarter of a fruit (accurate to 0.01 g) and was recorded as *W*1. The fruit was placed in the drying oven, baked at 105 °C for 1 h, and dried at 80 °C to constant weight, which was recorded as *W2*.

(1) Fruit moisture content=(W1−W2)/W1×100%

Parameters of tomato color including brightness (L*), redness or greenness (a*), and yellowness or blueness (b*) were measured as previously described using a Minolta Chroma Meter CR-10 Plus (Konica Minolta, Inc., Tokyo, Japan) [22].

The color index was calculated according to L*, a*, and b*, which was used to evaluate the tomato surface color index as a whole:

(2) $$Color\;index=2000\times \frac{a*}{L*}\times \sqrt[{\frac{1}{2}}]{{a*}^{2}+{b*}^{2}}$$

### 2.3. Determination of Soluble Solids, Vitamin C, and Lycopene

Tomato fruits with the same maturity and uniform size were selected for each variety, then quickly homogenized using a homogenizer to determine the content of soluble solids, vitamin C, and lycopene. The measurements were replicated three times with nine randomly selected fruits.

Firstly, the juice of a tomato fruit was dropped on a digital refractometer PAL-1 (Atago, Tokyo, Japan) and the value of the soluble solids were measured [23]. The vitamin C content of the fruits was measured using the 2,6-dichloroindophenol staining method in conjunction with a spectrophotometric measurement at 500 nm [24].

The tomato sample (0.5 g) was homogenized with a small amount of methylene chloride and then placed into a brown volumetric flask, and diluted with BHT dichloromethane solution (5 mg mL^−1^) to 25 mL. The sample was treated with an ultrasonic sonicator (Ningbo Scientz Biotech Company, Ningbo, China) at 35 °C, 40 KHz, for 30 min, and the extract was filtered through a 0.22 µm organic filter membrane. The lycopene content was determined by high-performance liquid chromatography (HPLC) using a C18 column (250 mm × 4.6 mm, 5 µm, Waters Symmetry). The mobile phase was 92:8 methanol: dichloromethane, the detection wavelength was 470 nm, the flow rate was 1.5 mL min^−1^, the column temperature was 30 °C, and the injection volume was 10 µL.

### 2.4. Analysis of Sugar and Acid Components

The homogenized tomato sample was ground with liquid nitrogen, and 0.5 g was accurately weighed and transferred to a 10 mL centrifuge tube. The sample was extracted using 5 mL of chromatographic ethanol (80%). The mixture was thoroughly shaken, and placed in a 35 °C water bath for 20 min for ultrasonic extraction. The extracts were centrifuged at 12,000 rpm for 15 min at room temperature. The total volume of supernatant was adjusted to 10 mL with 80% ethanol. Two milliliters of this mixture was dried using a vacuum centrifuge concentrator at 65 °C, and the residue was resuspended in 0.5 mL ultrapure water and 1 mL of acetonitrile, then filtered using a 0.22 µm organic phase microporous membrane [25]. The analysis of fructose and glucose was performed via a Waters Acquity Arc UHPLC system (Waters, MA, USA). The mobile phase consisted of 75% acetonitrile, 0.2% triethylamine, and 24.8% ultrapure water, the flow rate was 0.8 mL·min^−1^, and separation was performed using an XBridge BEH Amide column (4.6 × 150 mm, 2.5 μm). The injection volume was 10 µL, the detection wavelength was 254 nm, and the column temperature was 40 °C. The sugar content was determined according to the concentration curves of fructose and glucose standards (Yuanye Bio-Technology Co., Ltd., Shanghai China) [26].

The organic acid content in tomato fruit was analyzed according to the methods of a previous report [20]. A total of 1.5 g of tomato fruit powder was accurately weighed, extracted in 7.5 mL ultrapure water, and shaken at 950 rpm for 15 min, followed by centrifugation at 10,000 rpm for 10 min at 4 °C. Afterward, 2 mL of supernatant was filtered through a 0.22 μm membrane. The 20 µL filtrate was subsequently subjected to UHPLC (Waters, MA, USA) analysis on an Atlantis T3 column (4.6 × 150 mm, 3 µm).

The chromatographic separation used for organic acid detection employed NaH_2_PO_4_ (20 mM, pH 2.7) as the mobile phase, with a flow rate of 0.5 mL·min^−1^. The detection wavelength was 210 nm, and the column temperature was 30 °C. The organic acid contents were determined according to the concentration curves of citric acid, malic acid, tartaric acid, fumaric acid, α-ketoglutaric acid, and oxalic acid standards [23].

### 2.5. Sensory Profiling

Tomatoes without disease, insect pests, or mechanical damage, with the same maturity and uniform size, were selected and randomly presented for sensory evaluation after being cut. The interim review panel was composed of faculty and students (21–52 years of age) from the Horticulture College of Gansu Agricultural University that were previously trained in the quantitative description of tomato attributes. Appearance and flavor attributes were chosen to describe the fruit. Each descriptor was scored on a 5-point scale: the higher the score, the better the index [27]. Three sessions were undertaken in a sensory analysis laboratory, and 308 people participated in the evaluation.

### 2.6. Volatile Analysis

Tomato volatile analysis was performed as previously described [28,29], with minor variations. An 8.0 g aliquot of the fruit was weighed and quickly ground to a homogenized state, then the homogenate was put into a 15 mL screw-head headspace vial containing a magnetic stirring rotor. After that, 1.5 g Na_2_SO_4_ (used to disrupt the activity of endogenous enzymes) and 10 µL of 2-octanol (8.82 mg·L^−1^) standard sample were added. The vial was immediately sealed, placed on a constant temperature magnetic stirrer, and incubated at 50 °C at 500 rpm for 10 min. The volatile compounds were extracted by exposing a 50/30 µm DVB/CAR/PDMS SPME fiber (ANPEL Laboratory Technologies Inc., China) to the vial headspace for 30 min under continuous agitation. The fiber was manually inserted into the injection port and volatiles were desorbed for 5 min at 250 °C. Gas chromatography was performed on an HP-Innowax elastic quartz capillary column (60 m × 0.25 mm, 0.25 μm) at a constant flow of 1.0 mL·min^−1^ with helium as the carrier gas. The injector temperature was 250 °C. The oven temperature programming conditions were set at 40 °C for 2.5 min, with a 10 °C·min^−1^ ramp until 110 °C, then a 6 °C·min^−1^ ramp until 230 °C, and a final hold at 230 °C for 8 min. The mass spectrometer was operated by an electron impact (EI) method with an ionization energy of 70 eV and a source temperature of 230 °C. Mass spectra were acquired within a scanning range of 30 to 500 m/z.

Volatile compounds separated by GC-MS were analyzed by the mass spectrometry library (NIST 2014) according to their mass fragmentation pattern from the spectra database. Only compounds with an MS matching score above 80% were maintained. The concentration of each compound in the tomatoes was calculated by the internal standard method using the following formula [30]:

(3) The content of volatiles/(μg/kg)=(A1/A2)×(M1/M2)×1000 

where *A*1 and *A*2 are the component area of the detected composition and internal standard, respectively; *M*1 and *M*2 are the amounts of the internal standard and sample, respectively.

### 2.7. Calculation of Comprehensive Membership Function Value

The membership function value of each nutritional index was calculated using the following formula, where *Xij* (*u*) is the membership function value of the jth index of the ith variety, *Xij* is the measured value of the jth index of the ith variety, *Xjmax* is the maximum value of the jth index in the tested variety, and *Xjmin* is the minimum value of the jth index in the tested variety [31].

(4) Xij(u)=(Xij−Xjmin)/(Xjmax−Xjmin)

(5) Xij(u)=1−(Xij−Xjmin)/(Xjmax−Xjmin)

The value of the comprehensive membership function was calculated as follows:

(6) $$Xi\left(u\right)=[\sum_{i=1}^{n}Xij(u)]/n$$

### 2.8. Statistical Analysis

All data are reported as the mean ± standard error for three replicates. The Student’s *t*-test was used for comparison between different treatments using SPSS version 19.0 (IBM, Chicago, IL, USA). A difference was considered to be statistically significant when *p* < 0.05. The Origin 2021 software was used to generate figures and carry out principal component analysis (PCA).

## 3. Results

### 3.1. Physical Indexes of Tomato Fruit

Table 1 shows the fruit shape index, firmness, and moisture content of the 15 tomato varieties. From the fruit appearance, the shape index of Y_5_ was significantly higher than that of other varieties, which were obround. For other varieties, the fruit shape index was in the range 0.76–0.97, which is oblate or nearly circular. The firmness of regular tomato with or without peel was 32.75% and 157.82% higher than that of tasty tomato, and 36.09% and 54.50% higher than that of cherry tomato, respectively. The T_4_ tomato had the least firmness and was softest of all. The moisture content of the 15 tomato varieties was between 90.92% and 96.16%, with that of regular tomato being 3.16% and 1.90% higher than tasty and cherry tomato, respectively.

### 3.2. Color Parameters of the Tomato Surface

**Figure 2:**
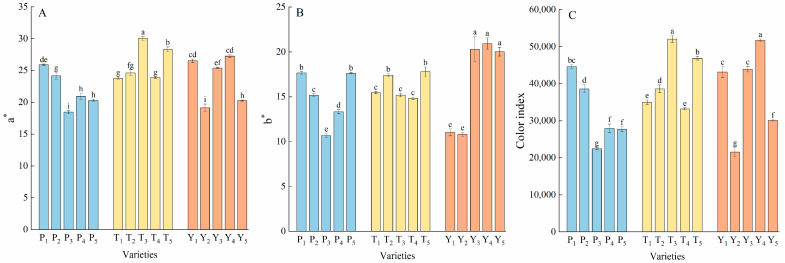
The a* values (**A**), b* values (**B**), and color indexes (**C**) of the tomatoes. Data represent the mean ± SE. Different lowercase letters indicate statistical significance by Duncan’s multiple range test (*p* < 0.05). P, regular tomatoes; T, tasty tomatoes; Y, cherry tomatoes.

The color parameters of the 15 varieties were found to differ significantly (Figure 2). The T_3_ and T_5_ varieties had the highest a* values (positive value represents red) among all varieties. The b* values (positive value means yellow) of the Y_3_, Y_4_, and Y_5_ varieties were the highest among all varieties. The average color value of tasty tomato was 8.03% higher than that of cherry tomato and 27.43% higher than that of regular tomato, while that of cherry tomato was 17.95% higher than that of regular tomato (Figure 2C).

### 3.3. Content of Soluble Solids, Vitamin C, and Lycopene in Tomato Fruit

**Figure 3:**
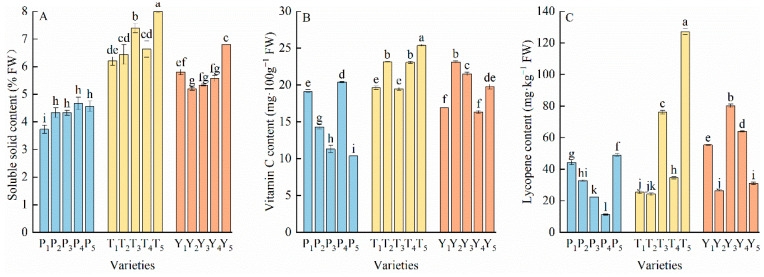
The soluble solids (**A**), vitamin C (**B**), and lycopene (**C**) contents in tomato fruits. Data represent the mean ± SE. Different lowercase letters indicate statistical significance by Duncan’s multiple range test (*p* < 0.05). P, regular tomatoes; T, tasty tomatoes; Y, cherry tomatoes.

The soluble solid content also differed among the different types of tomato fruits (Figure 3A). Overall, the content in the five tasty varieties was significantly higher than the regular tomato varieties, while that of cherry tomato was in between the other two types. P_1_ (3.73%) had the lowest soluble solid content among all varieties, while T_5_ (8%) had the highest value. The average vitamin C content in the five tasty tomato varieties was 46.46% higher than that of regular tomato and 13.33% higher than that of cherry tomato (Figure 3B); T_5_ had the highest vitamin C content (up to 25.39 mg·100g^−1^), while P_5_ had the lowest vitamin C content (10.38 mg·100g^−1^) among all varieties. The lycopene content of different varieties greatly varied, despite all 15 varieties being red tomatoes (Figure 3C). The T_5_, Y_3_, T_3_, Y_4_, and Y_1_ varieties had lycopene contents greater than 50 mg·kg^−1^. The average lycopene content of tasty tomato was 11.58% higher than that of cherry tomato and 79.96% higher than that of regular tomato.

### 3.4. Sugar and Organic Acid Content of Tomato Fruit

**Figure 4:**
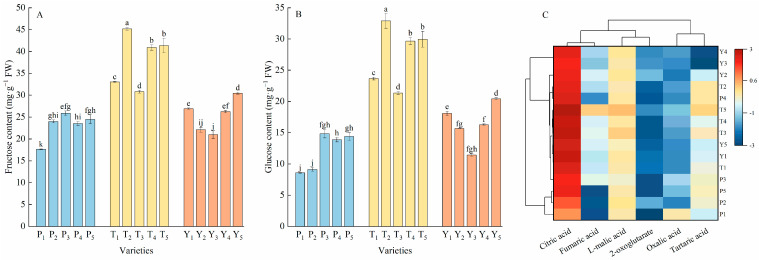
The contents of fructose (**A**), glucose (**B**), and organic acids (**C**) in tomato fruits. Data in (**A**,**B**) represent the mean ± SE. Different lowercase letters indicate statistical significance by Duncan’s multiple range test (*p* < 0.05). The colored areas in (**C**) correspond to the content of acids from high (red) to low (blue). The data were log2 transformed and standardized. Euclidean distance and average linkage were used to construct the clustering of acids and varieties. P, regular tomatoes; T, tasty tomatoes; Y, cherry tomatoes.

The soluble sugar and organic acid content constitute the soluble solids of tomato and accounted for 60% of the dry weight [32]. Fructose and glucose, the two soluble sugars, accounted for a large proportion of the tomato fruit. The fructose content of the 15 tomato varieties was between 17.65–45.18 mg·g^−1^ FW; this was slightly higher than the glucose content (Figure 4A,B). The average fructose and glucose contents in the five tasty varieties were 65.60% and 126.22% higher than those of regular tomatoes, and 50.96% and 67.96% higher than those of cherry tomatoes, respectively.

Six organic acids, namely, citric, malic, tartaric, fumaric, α-ketoglutaric, and oxalic acids, were detected in this study. The total organic acid content of tasty tomato was 40.89% higher than that of regular tomato and 25.63% higher than that of cherry tomato (Supplementary Table S1). As can be seen from Figure 4C, five regular tomato varieties are clustered together. Citric acid, the main organic acid in these tomato samples, accounted for 41.07–67.41% of the total organic acid content. L-malic acid is the second most abundant organic acid in the 15 varieties; it accounted for 11.67–20.97% of the total organic acid content. The sum content of oxalic acid, tartaric acid, fumaric acid, and α-ketoglutaric acid accounted for 16.58–38.12% of the total organic acids. Among them, the average oxalic acid content of regular tomato is 55.16% higher than that of tasty tomato and 90.67% higher than that of cherry tomato.

### 3.5. The Volatile Compounds in Tomato Fruit

**Figure 5:**
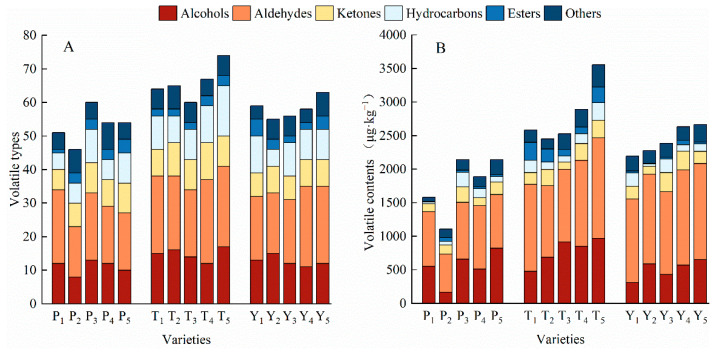
The types (**A**) and contents (**B**) of volatile compounds in tomato fruits. P, regular tomatoes; T, tasty tomatoes; Y, cherry tomatoes.

A total of 110 volatile compounds (including 31 alcohols, 33 aldehydes, 13 ketones, 16 hydrocarbons, 6 esters, and 11 other compounds) were detected in the 15 different tomato varieties using headspace solid-phase microextraction–gas chromatography–mass spectrometry (HS-SPME/GC-MS). Other compounds present in small quantities mainly included furans, phenols, thiazoles, and acids, among others (Supplementary Table S2). A total of 64–74 volatile compounds were detected in the tasty tomato varieties, with T_5_ containing the most (74), while cherry and regular tomato varieties contained 55–63 and 46–60, respectively (Figure 5A). The relative content of volatile compounds was estimated by adding certain amounts of internal standard substances to evaluate the differences in volatile compounds in the tomato fruits. P_2_ had the lowest total volatile compound content of the 15 tomato varieties (1109.06 μg·kg^−1^), while T_5_ had the highest content (3556.12 μg·kg^−1^) (Figure 5B). The average content of total volatile compounds in tasty tomato (2802.64 μg·kg^−1^) was 57.94% higher than that of regular tomato and 15.24% higher than that of cherry tomato. Aldehydes and alcohols were the two main volatile compounds in all the tested tomato varieties, and the sum of the two accounted for over 50% of the total volatile compounds.

### 3.6. Analysis of Characteristic Aroma Components of Tomato Fruit

The odor activity value (OAV) was calculated according to the quantitative results and their respective thresholds to determine the aroma contribution of volatile compounds in the 15 tomato varieties. The 34 aroma active components with an OAV greater than 1 were the key aroma components of tomato varieties in this study (Table 2). There were 18–25, 22–29, and 19–24 kinds of characteristic aroma components in regular, tasty, and cherry tomatoes, respectively. There were differences in the contents of characteristic aroma components and OAV values among different varieties that led to subtle differences in aroma presentation between tomato varieties.

Regular tomatoes had 10 characteristic aroma compounds shared by five varieties (P_1_–P_5_): linalool, β-ionone, decanal, hexanal, (Z)-3-hexenal, (E)-2-hexenal, 2-isobutylthiazole, 3-methylbutanal, (E)-2-octenal, and 1-octen-3-one. There were 20 characteristic aroma compounds shared in tasty tomatoes (T_1_–T_5_). Tasty tomatoes had no linalool, but contained 1-hexanol, 2,4-decadienal, (E,E)-2,4-Decadienal, ethyl ethanoate, 2-amylfuran, 2-hexanal, (Z)-3-hexen-1-ol, 1-octanol, 1-nonnol, (E)-2-decenal, and 1-octen-3-ol, unlike regular tomatoes. Cherry tomatoes had 10 common characteristic aroma compounds, and contained no linalool, decanal, or (Z)-3-hexanal 1-octen-3-one compared with regular tomatoes; but had 1-hexanol, methylheptenone, 2-hexanal, and (E)-2-heptenal.

**Figure 6:**
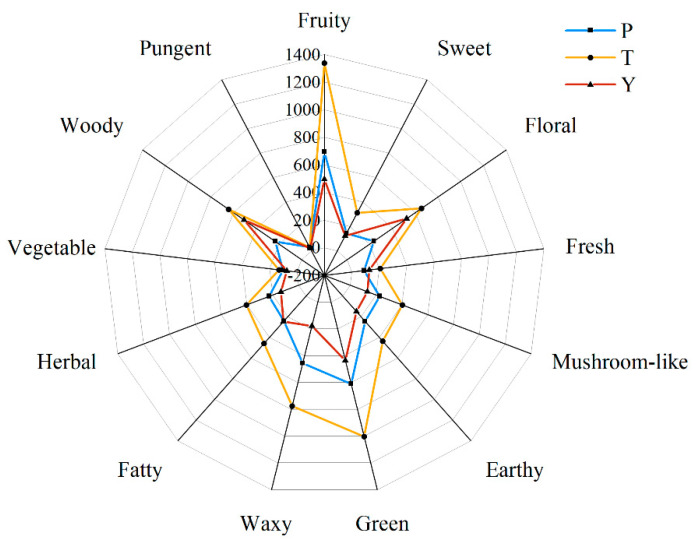
The aroma shape of the three types of tomato based on the characteristic aroma components with the odor activity values (OAVs). P, regular tomatoes; T, tasty tomatoes; Y, cherry tomatoes.

The 34 characteristic aroma compounds were divided into 13 types, fruity, sweet, floral, fresh, mushroom-like, earthy, green, waxy, fatty, herbal, vegetable, woody, and pungent OAV, which were used to draw aroma contour maps for the three types of tomato. The OAV for each aroma type were the same when a compound contributed to multiple aroma systems (Figure 6). Overall, the aromas of tasty tomato were similar to those of regular tomato, with fruity and green being the dominant aroma types, and other aroma types, except pungent, being more intense than regular tomatoes. Woody, floral, and green were the main aroma types of cherry tomato, and their intensity was similar to tasty tomato. All aroma types showed a higher value in tasty tomato compared to regular and cherry tomatoes, except for the pungent type.

### 3.7. Sensory Evaluation of Tomato Appearance and Flavor

There were significant differences between assessors in their ratings of appearance and flavor (Table 3), as is frequently found in sensory studies. The appearance evaluation scores of tasty, cherry, and regular tomatoes were calculated to be 3.44–4.08, 3.68–3.81, and 2.76–4.26, respectively, with the P_4_ variety assessed as the top variety. The T_5_ variety exhibited the highest score in terms of flavor. The flavor scores of the tasty tomatoes were in the range 4.31–4.86, and their average value was 17.32% higher than that of cherry tomato and 31.58% higher than that of regular tomato.

### 3.8. Correlation Analysis between Tomato Quality Index and Sensory Evaluation

**Figure 7:**
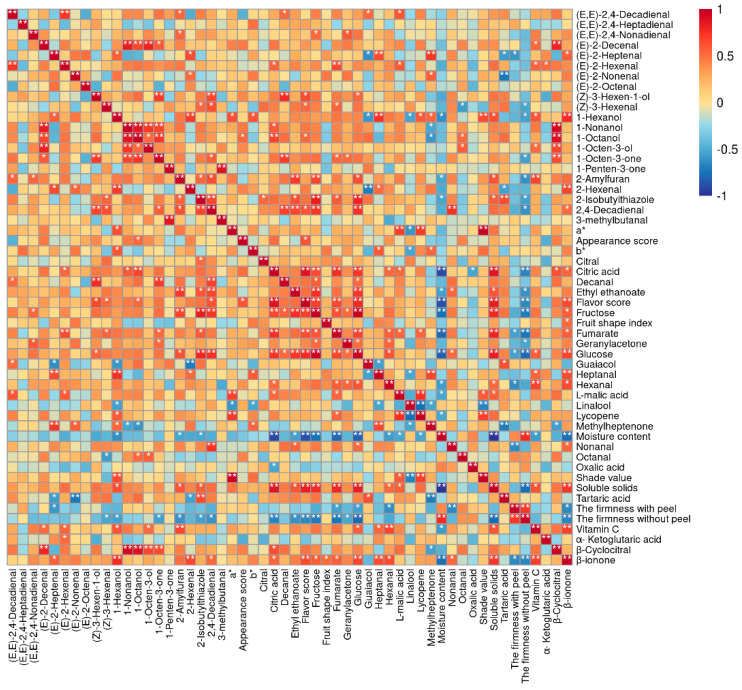
The heat map of Pearson’s correlation analysis. Values are Pearson’s correlation coefficients. * and ** denote correlation coefficients that are significant at the *p* < 0.05 and *p* < 0.01 levels, respectively.

Pearson’s correlation coefficient analysis showed linear relationships between the sensory scores and physicochemical properties of tomato samples. There was a significant negative correlation between moisture content and soluble solids and citric acid (r < −0.9, *p* < 0.01) (Figure 7). The flavor sensory score positively correlated with the content of soluble solids, citric acid (r > 0.8, *p* < 0.01) and glucose, (Z)-3-hexen-1-ol, 2,4-decadienal, and β-ionone (r > 0.6, *p* < 0.05); but negatively correlated with moisture content (r < −0.8, *p* < 0.01) and hardness without peel (r < −0.6, *p* < 0.05). Additionally, there were positive correlations between the flavor sensory score and the appearance sensory score of tomato (r = 0.60, *p* < 0.05).

### 3.9. Tomato Classification Model Using Principal Component Analysis

**Figure 8:**
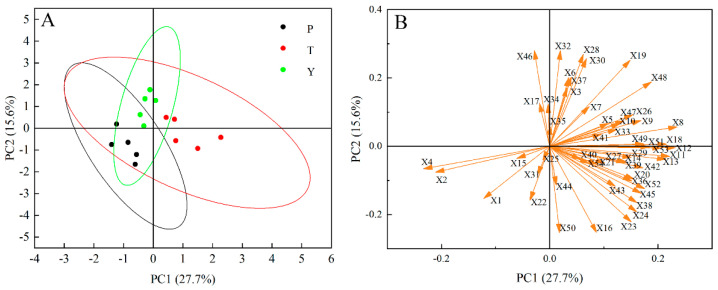
Principal component analysis (PCA) of tomato samples with a different score plot and loading plot. Circles in (**A**) are 95% confidence ellipses. The arrow in (**B**) indicates the loadings. P, regular tomatoes; T, tasty tomatoes; Y, cherry tomatoes.

A single index cannot accurately reflect tomato fruit quality. Principal component analysis (PCA) was used to comprehensively analyze multiple character indexes to improve the accuracy of the experiment (Figure 8a). The variance contribution rates of the first and second components (PC1 and PC2) were 27.7% and 15.6%, respectively. All regular tomatoes were located on the negative half of the *x*-axis, while all tasty tomatoes were located on the positive half of the *x*-axis, and all cherry tomatoes were located on the positive half of the *y*-axis. Tasty, cherry, and ordinary tomatoes were well distinguished on PC1 and PC2. Figure 8b shows the load value of each index in its main component. Soluble solid content (X8), glucose (X12), fructose (X11), citric acid (X13), moisture content (X4), and firmness without peel (X2) significantly contribute to PC1. This mainly shows the taste-related tomato indicators. (E)-2-heptenal (X32), methylheptenone (X46), heptanal (X28), guaiacol (X50), and tartaric acid (X16) make the greatest contribution to PC2. This primarily represents indicators related to tomato odor and taste. 3-methylbutanal (X25) has little effect on the differentiation of tomato types.

### 3.10. Calculation of Comprehensive Membership Function Value

The membership function method is one of the important methods for objective analysis and evaluation of food quality based on the determination of multiple indicators. The specific characteristic membership values of various indicators of different varieties of tomato were accumulated and the average value was obtained. Higher average values of the membership function correlated with improved variety quality. The final quality ranking was determined by comparing the average values of the membership functions of 53 quality-related indicators of 15 tomato materials (Table 4). The average values of the membership functions of tasty tomato, cherry tomato, and regular tomato were 0.42, 0.23, and 0.12. This indicates that the quality of tasty tomato was better than that of cherry tomato, and the quality of cherry tomato was better than that of regular tomato.

## 4. Discussion

### 4.1. Differences in Tomato Fruit Quality

In recent years, the decisive substances related to tomato flavor have been gradually identified. Tieman et al. [1] spent five years organizing 100 people to conduct multiple sensory properties tests on 150 varieties of tomato fruits to reveal the material basis of tomato flavor; 29 volatile substances related to consumer preferences were identified, and 37 substances were found to be significantly related to flavor intensity. Consumers liked the following flavor substances: soluble solids, citric acid, malic acid, glucose, fructose, geranylacetone, 6-methyl-5-hept-2-one, β-ionone, β-cyclocitral, geranyl aldehyde, linalool, 1-penten-3-one, (E)-3-hexen-1-ol, hexanal, heptaldehyde, (E)-2-heptylenal, 2-octenal, benzaldehyde, 2-phenylethanol, phenylacetaldehyde, phenyldialdehyde, and 2-isobutylthiazole, among others [33,34]. This study determined the key factors affecting the quality difference between three types of tomato fruit: regular, tasty, and cherry. A correlation analysis showed that citric acid content had the highest correlation coefficient among the indicators positively correlated with flavor score, while water content had the highest correlation coefficient among the negative correlation indicators. Therefore, it was inferred that the most important factors causing high flavor scores of tasty tomatoes were higher citric acid content and lower water content [35,36]. The correlation analysis results also showed that soluble solids and moisture content were negatively correlated (r > 0.9, *p* < 0.01). One explanation is that the low content of soluble solids in tomatoes is owing to the dilution effect caused by high fruit moisture content [37]. However, the correlation between appearance sensory scores and various indicators in this study was relatively low. This indicated that consumers’ preferences for tomato appearance were more subjective and difficult to evaluate using current indicators. Principal component analysis showed that regular and tasty tomatoes were mainly distinguished along the *x*-axis, and the key indicators were soluble solids, glucose, fructose, citric acid, moisture content, and firmness without peel. Cherry tomatoes were mainly distinguished from regular and tasty tomatoes along the *y*-axis, with key indicators being (E)-2-heptenal, methylheptenone, heptanal, guaiacol, and tartaric acid.

Some studies show that the tomato fruit flavor quality and the perceived sugar and acidity have a great impact on the taste [11], with sugar content being positively correlated with taste [38]. Higher sugar gives people a sense of taste pleasure, while moderate acidity can enhance the flavor [16,39]. This study detected two kinds of sugar (fructose and glucose) and six organic acids (citric, malic, tartaric, fumaric, α-ketoglutaric, and oxalic acids) in tomato, among which citric acid accounted for over 41% of the total acid content. The total organic acid and citric acid contents of tasty tomato were higher than those of regular tomato and cherry tomato. It is worth noting that the oxalic acid content of regular tomatoes is higher than those of tasty tomatoes and cherry tomatoes. However, oxalic acid reduces the calcium utilization rate, and may increase the risk of kidney stones in the human body [40]. A high oxalic acid content is an adverse factor for regular tomatoes from the perspective of human health. Some studies have shown that the sugar, soluble solid, lycopene, and vitamin C content of cherry tomatoes are higher than those of regular tomatoes [41,42]. The cherry tomato and regular tomato varieties in this study showed similar characteristics. However, tasty tomatoes had the characteristics of lower relative water content, lower fruit hardness, and significantly higher contents of soluble solids, vitamin C, and lycopene compared with regular and cherry tomatoes, making them more popular among consumers. Tomatoes’ physical characteristics (such as taste firmness) have an impact on fruit quality, merchantability, and shelf life. Regular tomatoes have a higher firmness, possibly owing to breeders focusing on long-term domestication and improvement to enhance economic traits such as tomato yield and resistance [43]. These varieties have insufficient flavor and aroma. In general, the sensory score of tasty tomato was significantly higher than those of cherry and regular tomatoes. This indicates that tasty tomatoes have broad market prospects.

### 4.2. Aroma Characteristics of Tomato Fruit

Aroma is mainly composed of volatile aromatic compounds in the fruit that determine the unique flavor of tomato [15,44]. Tomatoes contain over 400 volatile aromatic compounds [19]. Some studies show that the different types, proportions, and balance of volatile compounds in the metabolic components of tomato fruit lead to differences in the taste of different tomato varieties [45]. This study identified a total of 110 volatile compounds. Both in terms of quantity and content, alcohols and aldehydes were the most abundant two categories, which is consistent with the results of Selli S. et al.’s study on cherry tomatoes [46]. The odor characteristics of volatiles with different functional groups greatly differ [47]. Aldehydes account for 29.73% of the volatile compounds identified in this study; they have a grass flavor and can increase the freshness of tomatoes [48,49]. Alcohols, accounting for 27.93%, have a sweet taste, and play an important role in improving tomato flavor [50]. Ketones, with floral, fruity, and sweet-smelling odors, account for 11.71%; these smells are preferred by people [51]. The ester content in mature tomato fruit is very low, and the smell is disliked by people [52]. In this study, the average volatile compound content of tasty tomato was 57.94% higher than that of regular tomato and 15.24% higher than that of cherry tomato. This indicates that tasty tomatoes have more advantages in odor characteristics.

The OAV is usually applied to evaluate the contributions of certain aroma compounds; aroma compounds with OAVs > 1 are generally considered to make an important contribution to the overall aroma characteristics [53]. No more than 30 of the identified volatile compounds play a major role in 174 tomato accessions (comprised of 123 cherry tomato accessions (*Solanum lycopersicum var. cerasiforne*) and 51 large-fruit cultivators (*S. lycopersicum*)) flavor [54]. Cheng, G. et al. showed that tomato odor was mainly derived from 15 volatiles [13]. Tang et al. revealed that the flavor components leading to consumer approval were 10 volatile compounds including decanal, 2-isobutylthiazole, and β-ionone [55]. This study showed that 34 aroma-active compounds constitute the aroma of the three types of tomato, including six alcohols, 19 aldehydes, five ketones, and four other substances. Among them, three compounds had OAV values below 1 or were not detected in regular tomatoes: (E)-2-nonenal, (E,E)-2,4-nonadienal, and 2-amylfuran. The OAV value of citral was below 1 in most tomato varieties except one regular tomato and one cherry tomato. (E,E)-2,4-decadienal was not detected in two regular tomato and one cherry tomato varieties. The OAVs of five volatile compounds were greater than 1 in all tomato varieties, including hexanal, (E)-2-hexenal, (E)-2-octenal, β-ionone, and 2-isobutylthiazole. Hexanal, (E)-2-hexenal, and (E)-2-octenal provide a fresh and green smell for tomatoes, and their precursors were all from the fatty acid pathway. β-Ionone is synthesized from carotenoid decomposition and gives tomato a floral and woody flavor [56]. Despite its extremely low odor threshold, it is very important for tomato aroma formation. There was a significant correlation between β-ionone and flavor score. The mean OAV of β-ionone in tasty tomato was 179.95% higher than that of regular tomato and 8.08% higher than that of cherry tomato. 2-Isobutylthiazole is a unique aromatic substance of tomato that provides tomato fruit and green flavor [57]. The average OAV value of 2-isobutylthiazole in tasty tomato was 74.58% higher than that of regular tomato and 121.51% higher than that of cherry tomato.

Du, X. et al. indicated that the most intense aroma category was earthy–musty, followed by fruity–floral and green–grassy in two Florida tomato varieties [58]. In the present study, tasty tomato had the most intense aromas, that mainly consisted of fruity and green aromas; these aromas were similar to those of regular tomato. Meanwhile, cherry tomato was mainly composed of floral and woody aromas. (E)-2-heptenal, methylheptenone, heptanal, and guaiacol were the main reasons for the different odors of cherry tomatoes compared to regular tomatoes and tasty tomatoes from the perspective of PCA. Correlation analysis showed that some volatile compounds were significantly correlated with one other, and there is a certain correlation with other quality indicators. It is speculated that they may interact and affect the flavor quality of tomato fruit. However, further research is required to determine how this interaction occurs.

## 5. Conclusions

Tasty tomatoes have lower hardness and moisture content, and higher contents of soluble solids, vitamin C, lycopene, and fructose compared with regular and cherry tomatoes. The average volatile compound content of tasty tomatoes was 57.94% higher than that of regular tomato and 15.24% higher than that of cherry tomato. Nineteen and ten characteristic aroma components were identified in tasty and regular tomatoes, respectively, with fruity and green aromas dominating the aroma types. Meanwhile, 10 characteristic aroma components were identified in cherry tomatoes, with the dominant being floral and woody aroma types. Comprehensive analysis can distinguish between the three types of tomatoes; the quality of tasty tomatoes was better than cherry tomatoes, followed by regular tomatoes. The flavor of the tomatoes was positively related to soluble solids, fructose, glucose, citric acid, fumaric acid, and β-ionone (*p* < 0.01), and showed a significant negative correlation with moisture content and hardness without peel.

## Figures and Tables

**Table 1:** Physical properties of tomato fruits.

Variety	Firmness with Peel (kg·cm^−2^)	Firmness without Peel (kg·cm^−2^)	Fruit Shape Index	Moisture Content (%)
P_1_	3.37 ± 0.08 b	2.14 ± 0.08 a	0.82 ± 0.01 def	96.16 ± 0.01 a
P_2_	3.53 ± 0.04 b	1.99 ± 0.03 a	0.85 ± 0.01 cde	95.80 ± 0.02 b
P_3_	2.50 ± 0.05 de	1.19 ± 0.08 c	0.76 ± 0.03 f	95.25 ± 0.06 c
P_4_	4.36 ± 0.15 a	2.18 ± 0.10 a	0.82 ± 0.01 def	94.72 ± 0.04 d
P_5_	3.50 ± 0.09 b	1.56 ± 0.05 b	0.80 ± 0.01 ef	94.51 ± 0.05 e
T_1_	2.38 ± 0.05 de	0.70 ± 0.01 de	0.76 ± 0.02 f	93.19 ± 0.07 j
T_2_	2.43 ± 0.15 de	0.81 ± 0.07 d	0.97 ± 0.03 b	93.04 ± 0.03 k
T_3_	3.63 ± 0.08 b	0.82 ± 0.04 d	0.78 ± 0.02 f	92.00 ± 0.04 n
T_4_	2.05 ± 0.05 f	0.59 ± 0.03 e	0.85 ± 0.01 cde	92.70 ± 0.03 l
T_5_	2.49 ± 0.04 d	0.59 ± 0.03 e	0.88 ± 0.01 cd	90.92 ± 0.03 o
Y_1_	2.48 ± 0.15 d	1.12 ± 0.06 c	0.79 ± 0.02 ef	93.45 ± 0.02 i
Y_2_	2.16 ± 0.17 ef	1.04 ± 0.07 c	0.90 ± 0.01 c	94.23 ± 0.00 f
Y_3_	3.01 ± 0.06 c	1.09 ± 0.03 c	0.87 ± 0.01 cd	93.89 ± 0.04 g
Y_4_	2.56 ± 0.13 d	1.16 ± 0.07 c	0.89 ± 0.01 c	93.74 ± 0.04 h
Y_5_	2.47 ± 0.01 d	1.45 ± 0.14 b	1.30 ± 0.05 a	92.23 ± 0.02 m

Note: data represent the mean ± SE. Different lowercase letters indicate statistical significance by Duncan’s multiple range test (*p* < 0.05). P, regular tomatoes; T, tasty tomatoes; Y, cherry tomatoes.

**Table 2:** Volatile compounds with odor activity value ≥ 1 in tomato fruits.

Characteristic Aroma Components	CAS Number	Aroma Type	Odor Activity Values (OAVs)															Odor Threshold
			P_1_	P_2_	P_3_	P_4_	P_5_	T_1_	T_2_	T_3_	T_4_	T_5_	Y_1_	Y_2_	Y_3_	Y_4_	Y_5_	μg/kg
1-Hexanol	111-27-3	Fruity, sweet, green	12.51	0.00	0.00	0.00	0.00	14.33	12.60	14.34	10.53	19.63	8.51	9.72	17.47	20.91	14.87	5.7
(Z)-3-Hexen-1-ol	928-96-1	Fresh, green	2.26	0.68	2.54	1.76	3.44	1.56	2.68	3.65	3.38	3.68	0.83	1.85	1.15	2.06	2.34	200
1-Octen-3-ol	3391-86-4	Mushroom, earthy, green	3.81	2.41	6.16	16.22	0.00	7.73	5.16	6.23	12.78	8.48	1.50	4.82	14.61	5.42	4.84	1
Linalool	78-70-6	Citrus, floral, sweet	1.94	1.81	2.96	3.55	2.29	2.92	2.13	2.30	3.46	0.00	2.36	2.47	0.00	0.00	3.57	1.5
1-Octanol	111-87-5	Waxy, green, citrus,	0.00	0.00	471.45	1017.08	314.78	244.55	200.02	580.71	1054.24	919.37	389.87	224.69	0.00	286.38	0.00	0.023
1-Nonanol	143-08-8	Fresh, fatty, floral	1.26	0.00	2.34	5.44	0.00	1.77	1.39	4.26	6.57	5.21	1.93	0.00	2.57	0.00	0.00	2
3-methylbutanal	590-86-3	Fruity, fatty	17.41	31.66	37.71	32.56	25.19	50.50	21.14	16.13	26.04	23.36	31.00	0.00	49.45	14.66	22.97	0.25
Hexanal	66-25-1	Fresh, green, fruity	30.34	14.30	28.52	36.39	22.55	24.41	54.35	29.02	55.99	57.52	49.46	58.77	30.72	48.27	47.15	4.5
(Z)-3-Hexenal	6789-80-6	Green, fatty, fruity	24.27	46.05	141.86	40.52	108.54	161.84	104.91	97.07	70.27	133.75	102.18	106.87	48.92	0.00	124.17	0.25
Heptanal	111-71-7	Fresh, green, herbal	1.48	0.00	0.00	0.00	0.00	1.80	1.83	0.00	0.00	2.01	0.00	1.69	2.69	1.65	1.75	2.9
(E)-2-Hexenal	6728-26-3	Green, banana	5.02	10.30	7.50	10.05	5.20	10.05	7.27	7.02	10.61	15.01	8.50	10.19	9.00	8.51	8.34	40
2-Hexenal	505-57-7	Sweet, fruity, green	20.95	0.00	16.41	12.80	21.20	28.34	19.40	29.97	18.87	22.37	29.51	30.46	32.99	34.03	36.89	17
Octanal	124-13-0	Citrus, green, waxy	3.16	0.00	0.00	23.15	0.00	0.00	0.00	0.00	8.10	0.00	0.00	2.59	0.00	12.20	0.00	0.7
(E)-2-Heptenal	18829-55-5	Pungent, green, fresh	1.49	1.11	1.56	0.49	0.98	5.35	2.34	0.92	1.53	1.40	1.92	3.68	2.94	4.79	3.89	13
Nonanal	124-19-6	Waxy, fresh, citrus	13.70	0.00	11.28	0.00	5.84	18.26	11.72	0.00	15.25	17.38	0.00	11.72	0.00	9.13	10.00	1
(E)-2-Octenal	2548-87-0	Fresh, green, herbal	2.77	2.84	1.60	13.09	3.10	14.30	10.35	5.08	2.87	2.54	14.96	6.33	6.28	11.09	10.21	3
(E,E)-2,4-Heptadienal	4313-03-5	Fatty, green, vegetable	46.26	95.31	0.00	72.99	266.39	141.03	217.30	193.93	0.00	51.55	0.00	0.00	31.82	203.56	118.01	0.08
Decanal	112-31-2	Sweet, waxy, citrus	73.32	76.22	72.04	53.73	90.50	90.78	68.85	125.75	159.20	133.00	0.00	47.72	0.00	95.87	82.31	0.1
(E)-2-Nonenal	18829-56-6	Fatty, green, citrus	0.00	0.00	0.00	0.00	0.00	0.00	0.00	0.00	11.31	0.00	7.51	0.00	9.33	16.67	12.63	0.4
β-Cyclocitral	432-25-7	Herbal, rose, sweet	0.82	0.00	0.44	8.18	0.53	0.56	0.41	7.06	11.28	7.67	2.00	0.33	2.55	1.39	0.58	5
(E)-2-Decenal	3913-81-3	Waxy, green, earthy	9.81	0.00	12.44	27.79	0.00	10.49	9.79	23.60	32.67	28.70	14.11	7.67	31.88	19.82	0.00	0.3
Citral	5392-40-5	Citral, sweet	0.60	0.30	1.39	0.00	0.00	0.52	0.48	0.72	0.63	0.59	0.46	0.00	0.00	0.00	1.44	5
(E,E)-2,4-Nonadienal	5910-87-2	Melon, waxy, green	0.00	0.00	0.00	0.00	0.00	0.00	65.06	0.00	58.39	0.00	0.00	0.00	45.38	0.00	0.00	0.09
2,4-Decadienal	2363-88-4	Sweet, fresh, citrus	49.13	0.00	81.92	0.00	101.69	162.93	121.52	75.07	175.65	132.02	0.00	47.04	0.00	0.00	79.59	0.07
(E,E)-2,4-Decadienal	25152-84-5	Fatty, melon, citrus	0.00	0.00	80.00	53.57	81.00	138.57	155.86	131.43	448.43	80.43	0.00	66.00	175.43	111.71	116.86	0.07
1-Penten-3-one	1629-58-9	Pungent	16.41	35.56	39.79	33.08	26.14	49.92	9.75	11.20	26.39	39.80	30.74	9.34	41.20	7.18	17.71	1
1-Octen-3-one	4312-99-6	Herbal, mushroom, earthy	62.46	88.42	449.91	206.16	307.47	231.10	193.40	367.06	630.53	553.93	0.00	111.45	200.29	322.79	0.00	0.003
Methylheptenone	110-93-0	Citrus, green	1.51	1.12	1.41	0.52	1.03	1.60	1.32	0.55	0.93	1.21	1.32	1.10	1.88	2.23	1.60	50
Geranylacetone	3796-70-1	Foral, fruity, green	0.00	0.00	1.34	0.48	1.00	0.00	2.05	0.53	1.77	1.77	1.00	0.36	1.64	2.07	0.79	60
β-ionone	79-77-6	Floral, woody	326.92	39.84	182.03	267.40	328.76	707.44	621.99	394.14	718.24	763.47	432.38	542.18	635.60	530.22	825.26	0.007
Ethyl ethanoate	141-78-6	Fruity sweet	0.00	4.63	2.08	1.14	1.14	26.02	18.94	8.98	7.61	20.72	0.34	0.16	1.23	5.16	0.45	10
Guaiacol	90-05-1	Woody	0.00	3.35	4.11	2.99	2.02	0.00	1.66	0.00	2.12	3.76	2.76	0.00	0.00	0.00	0.00	2
2-Amylfuran	3777-69-3	Fruity, green, earthy	0.82	0.99	0.00	0.00	0.00	1.77	1.67	1.48	2.18	2.13	0.00	0.54	1.42	0.00	0.39	4.8
2-Isobutylthiazole	18640-74-9	Green, earthy, vegetable	5.72	5.62	9.05	4.02	5.07	7.91	12.10	8.16	10.73	12.59	9.24	2.85	2.49	2.44	6.23	3

Note: P, regular tomatoes; T, tasty tomatoes; Y, cherry tomatoes.

**Table 3:** Sensory evaluation scores of tomato appearance and flavor.

Variety	Flavor Score	Appearance Score	Variety	Flavor Score	Appearance Score	Variety	Flavor Score	Appearance Score
P_1_	2.85	3.48	T_1_	4.52	3.44	Y_1_	3.89	3.81
P_2_	2.54	2.76	T_2_	4.31	3.74	Y_2_	3.47	3.68
P_3_	3.81	3.87	T_3_	4.79	4.08	Y_3_	3.52	3.70
P_4_	4.10	4.26	T_4_	4.48	3.73	Y_4_	4.02	3.70
P_5_	4.15	3.70	T_5_	4.86	3.65	Y_5_	4.67	3.80

Note: P, regular tomatoes; T, tasty tomatoes; Y, cherry tomatoes.

**Table 4:** Tomato membership function score and ranking.

Variety	Membership Function	Ranking	Variety	Membership Function	Ranking	Variety	Membership Function	Ranking
P_1_	0.14	12	T_1_	0.35	4	Y_1_	0.15	10
P_2_	0.07	15	T_2_	0.38	3	Y_2_	0.13	13
P_3_	0.15	11	T_3_	0.30	6	Y_3_	0.28	7
P_4_	0.20	9	T_4_	0.50	2	Y_4_	0.27	8
P_5_	0.10	14	T_5_	0.55	1	Y_5_	0.31	5

Note: P, regular tomatoes; T, tasty tomatoes; Y, cherry tomatoes.

## Data Availability

The data that support the findings of this study are available from the corresponding author upon reasonable request.

## Supplementary Materials

The following supporting information can be downloaded at: https://www.mdpi.com/article/10.3390/plants12162947/s1, Table S1: The contents of organic acids in tomato fruits; Table S2: The contents of volatile compounds in tomato fruits.
